# In Silico Modelling to Assess the Electrical and Thermal Disturbance Provoked by a Metal Intracoronary Stent during Epicardial Pulsed Electric Field Ablation

**DOI:** 10.3390/jcdd9120458

**Published:** 2022-12-14

**Authors:** Ana González-Suárez, Juan J. Pérez, Barry O’Brien, Adnan Elahi

**Affiliations:** 1 School of Engineering, University of Galway, H91 TK33 Galway, Ireland; 2 Translational Medical Device Lab, University of Galway, H91 YR71 Galway, Ireland; 3 BioMIT, Department of Electronic Engineering, Universitat Politècnica de València, 46022 Valencia, Spain; 4 AtriAN Medical Limited, Unit 204, University of Galway Business Innovation Centre, Upper Newcastle, H91 W60E Galway, Ireland

**Keywords:** cardiac arrhythmia, computer modelling, coronary artery, epicardial ablation, metal stent, pulsed field ablation

## Abstract

Background: Pulsed Electric Field (PEF) ablation has been recently proposed to ablate cardiac ganglionic plexi (GP) aimed to treat atrial fibrillation. The effect of metal intracoronary stents in the vicinity of the ablation electrode has not been yet assessed. Methods: A 2D numerical model was developed accounting for the different tissues involved in PEF ablation with an irrigated ablation device. A coronary artery (with and without a metal intracoronary stent) was considered near the ablation source (0.25 and 1 mm separation). The 1000 V/cm threshold was used to estimate the ‘PEF-zone’. Results: The presence of the coronary artery (with or without stent) distorts the E-field distribution, creating hot spots (higher E-field values) in the front and rear of the artery, and cold spots (lower E-field values) on the sides of the artery. The value of the E-field inside the coronary artery is very low (~200 V/cm), and almost zero with a metal stent. Despite this distortion, the PEF-zone contour is almost identical with and without artery/stent, remaining almost completely confined within the fat layer in any case. The mentioned hot spots of E-field translate into a moderate temperature increase (<48 °C) in the area between the artery and electrode. These thermal side effects are similar for pulse intervals of 10 and 100 μs. Conclusions: The presence of a metal intracoronary stent near the ablation device during PEF ablation simply ‘amplifies’ the E-field distortion already caused by the presence of the vessel. This distortion may involve moderate heating (<48 °C) in the tissue between the artery and ablation electrode without associated thermal damage.

## 1. Introduction

Pulsed Electric Field (PEF) ablation, also known as Pulsed Field Ablation (PFA), has been recently proposed to ablate cardiac ganglionic plexi (GP) aimed to treat atrial fibrillation (AF) [1,2,3]. The epicardial approach involves placing a multi-electrode ablation catheter on the epicardial fat, where the GPs are located. In a previous computational modelling study, we observed that due to fat having a lower electrical conductivity than the myocardium, the electric field (E-field) values higher than 1000 V/cm (i.e., PEF-zone) are mainly confined to epicardial fat, hardly affecting the myocardium [4]. We also found that the presence of the GP in the middle of the fat layer provoked a distortion of the E-field distribution (due to the nerve tissue being more electrically conductive than the surrounding adipose tissue), and a decrease in the E-field value at the GP. We now hypothesize that the presence of other tissues and materials even more conductive than neuronal tissue, such as blood inside a coronary artery or the presence of a metal coronary stent, could distort much more the E-field distribution even to the point of causing thermal side effects.

The presence of a metal stent during electroporation has been already experimentally and computationally studied by Hogenes et al. [5] in the context of tumour ablation with two needle electrodes (bipolar ablation). They observed a disturbance and redistribution of the E-field, along with a substantial reduction in E-field value, when a metal stent was placed near the needle electrodes. Another experimental study concluded that although the vicinity of a metal stent did not cause notable increased heating of the metal stent itself, higher temperature increase around the ablation electrodes was observed when the stent was present, suggesting removing the metal stents prior to electroporation whenever possible [6,7]. Given that there is evidence that the presence of a metal stent somehow disturbs the E-field distribution during tumour electroporation, it is necessary to explore this same point in the context of cardiac PEF ablation. For that, we planned a computer modelling study to assess how the E-field distribution is affected by the presence of a metal intracoronary stent, along with the possible thermal side effects. In this sense, we used Laplace’s Equation to solve the electrical problem, while the Bioheat Equation was used for the thermal problem. Note that other mathematical approaches have been proposed to solve the thermal problem in biological tissues subjected to thermal therapies, such as porous media theory [8]. In fact, this theory has been also applied to study the macromolecular transport within an artery with the presence of a stent [9].

## 2. Methods

### 2.1. Model Geometry

**Figure 1:**
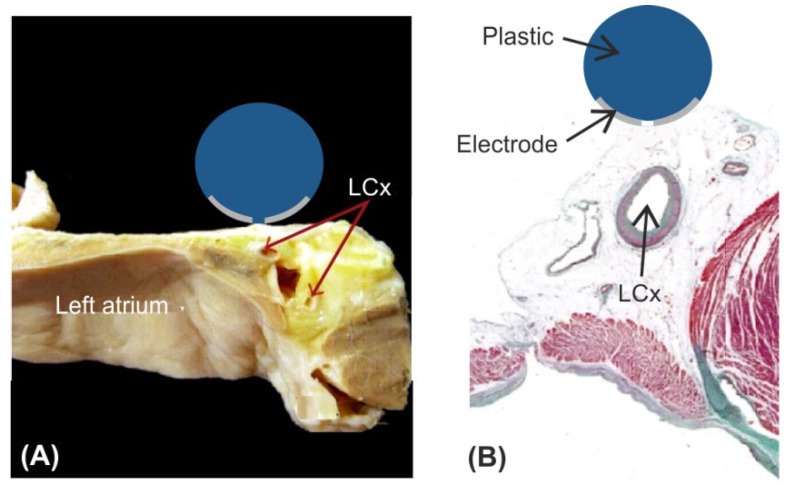
Physical situation modelled in the study. Anatomical picture (**A**) and histological image (**B**) showing the spatial relation between the ablation electrode and the left circumflex coronary artery –LCx–. Reprinted adapted with permission from [10]. Copyright © 2014 Damián Sánchez-Quintana et al. (it is an open access article distributed under the Creative Commons Attribution License (Attribution 3.0 Unported (CC BY 3.0)).

**Figure 2:**
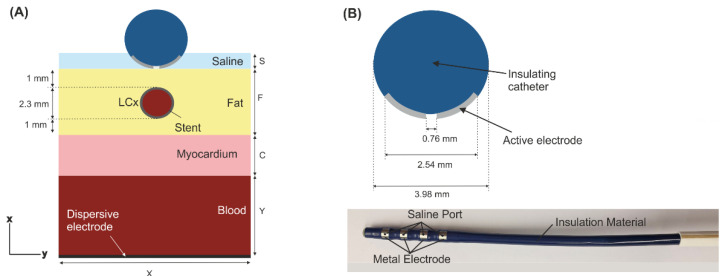
(**A**): Geometry of the 2D limited-domain model which only considers a fragment of the region of interest around the ablation device. The ablation device is embedded on a saline layer (S = 0.5 mm) and placed over an epicardial fat surface (F), myocardium (C = 2.7 mm) and blood layers. The metal stent is within the left circumflex coronary artery (LCx) with a wall thickness of 100 μm at 1 mm below the centre of the ablation device. The dispersive pad is placed on the bottom surface of the model. (**B**): Detail of the ablation device (AtriAN Medical, Galway, Ireland) with a hole in its centre for saline infusion [1,4,11].

Figure 1 shows the modelled physical situation in which an ablation electrode is placed on the epicardium with the left coronary artery (LCx) typically situated just below the ablation site (anatomical and histological pictures adapted from [10]). We considered a 2D limited-domain model including only the region of interest around the ablation device as shown in Figure 2. The validity of this approach has been previously demonstrated in comparison with a full torso model [11]. The model consisted of the ablation device placed over different layers (saline, fat, myocardium and blood). The dimensions of the ablation device were the same as a real device [1,4,11]: 3.98 mm diameter, metal electrode of 2.56 mm length with a 0.76 mm diameter irrigation hole in its centre. The metal electrode of the ablation device was assumed to be in direct contact with the epicardial fat surface and fully embedded in a saline layer of 0.5 mm thickness. This thin saline layer between the ablation electrode and the epicardium (target) acts as a ‘virtual electrode’, thereby ensuring the transmission of electrical energy to the target and preventing damage to the surrounding structures [1]. Myocardium thickness was of 2.7 mm [12] and the outer blood dimensions were similar to those of a limited-domain model of epicardial PEF ablation checked in a previous work by a sensitivity analysis, specifically X = 80 mm and Y = 40 mm [11].

In a real scenario, a stent is a hollow metal tube with a very thin wall between 60 to 140 μm [13]. The stent was thus modelled as a tubular metal cylinder of 100 μm wall inside the LCx of 2.3 mm diameter (which is within the reported range of 1.9–2.7 mm) [12]. The artery was assumed to be embedded in the epicardial fat, in the centre below the ablation device (see Figure 2). Two different distances between the artery and the ablation electrode were considered: 0.25 mm and 1 mm.

### 2.2. Governing Equations

The model was based on a coupled electrical-thermal problem, which was solved numerically by the Finite Element Method (FEM) with COMSOL Multiphysics (COMSOL, Burlington, MA, USA). A quasi-static approximation was employed for the electrical problem. The transient cellular responses were not considered (i.e., membrane charging), then the electric field distribution can be computed by solving Maxwell’s equations in its Laplacian form [14]:

(1) ∇· (σ∇ϕ)=0

(2) E=−∇ϕ

(3) J=σE

where σ is the electrical conductivity of the material, ϕ the electrical voltage, **E** the electric field vector, and **J** the current density vector.

The thermal problem was considered to evaluate any possible thermal side effect during epicardial PEF ablation. This problem was solved by using the Bioheat Equation [15]:

(4) $$\rho c\frac{\partial T}{\partial t}=\nabla \cdot \left(k\nabla T\right)+Q+Q_{p}+Q_{\mathrm{met}}$$

where ρ is density (kg/m^3^), c specific heat (J/kg·K), T temperature (°C), t time (s), k thermal conductivity (W/m·K), Q the heat source caused by the electrical power associated with PEF (W/m^3^) which is proportional to the electrical conductivity and to the square of the electric field magnitude Q = σ|**E**|^2^, Q_p_ the heat loss caused by blood perfusion (W/m^3^) and Q_met_ the metabolic heat generation (W/m^3^). Both Q_me_*_t_* and Q_p_ were ignored as these terms are negligible compared to the others [15].

### 2.3. Material Properties

The electrical and thermal properties (electrical conductivity, thermal conductivity, density and specific heat) of the model elements are shown in Table 1 [16,17,18,19,20]. The electrical conductivity increases during PEF ablation as the cell becomes more permeable to electrical current when PEF-induced pores are created. Previous experimental results showed that the best fit to model the change of electrical conductivity with the electrical field is achieved with a sigmoid function [21]. We also considered an increase of +2% of the electrical conductivity with the temperature [22] for myocardium, fat, blood and saline. Therefore, the electrical conductivity was modelled as follows:

(5) $$\sigma \left(E,T\right)=\left(\sigma_{0}+\frac{\sigma_{1}-\sigma_{0}}{1+10e^{-}\frac{\left(\left|E\right|-58,000\right)}{\begin{array}{c} 3000 \end{array}}}\right)\cdot 1.02^{T-37\;℃}$$

where σ_0_ and σ_1_ are the pre- and post-electroporation electric conductivities, respectively [11]. The values of σ_0_ and σ_1_ are related with the presence or lack of pores created in the cell membrane. Before PFA, i.e., when the pores are not still created, electrical current flows only through the extracellular material, in the same way that it does when the tissue is subjected to a low frequency electrical excitation and the cell membrane acts as an electrical insulator. For this reason, the values of σ_0_ were taken from measurement at low frequency, specifically that range of frequencies below beta dispersion. In practical terms, σ remains more or less constant between 1 and 10 kHz, and for higher frequencies decrease due the electrical current flowing not only through the extracellular material but also through the cytoplasm. According to this, we considered the pre- and post-electroporation conductivities at 10 Hz and 500 kHz (σ_0_ and σ_1_, respectively). The impact of selecting other pre- and post-electrical conductivities at other low and high-frequencies on the PEF-zone is negligible, as we confirmed in a previous sensitivity analysis [11].

### 2.4. Boundary Conditions

**Figure 3:**
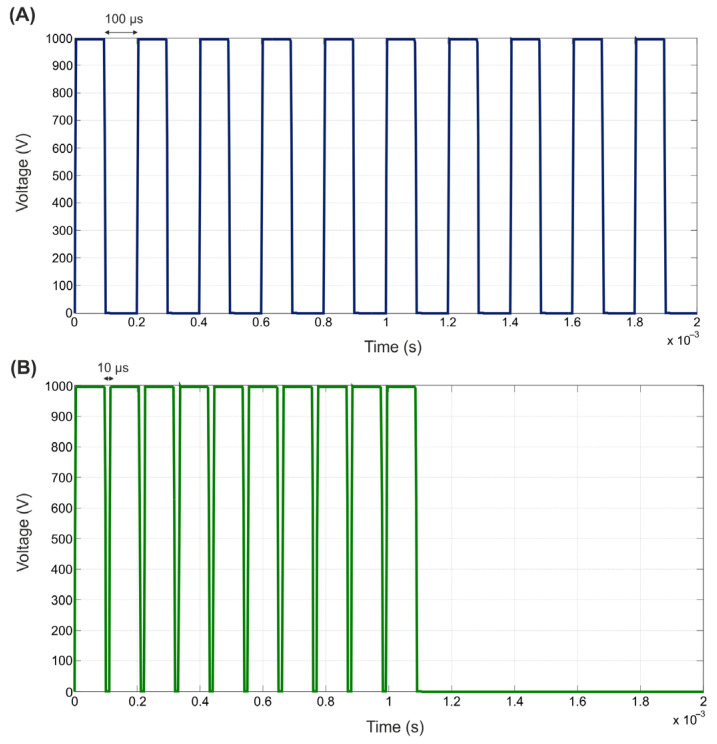
PEF pulse trains applied in the active electrode based on 10 consecutive pulses of 1000 V for 100 µs with an inter-pulse interval of 100 µs (**A**) and 10 µs (**B**).

Electrical and thermal boundary conditions were applied over the limits of the model. Regarding electrical boundary conditions, PEF ablation settings consisted of applying a pulse train based on 10 consecutive pulses of 1000 V for 100 µs each using a monopolar ablation mode, i.e., the energy was applied between the active electrode and the dispersive one (in the bottom surface of the model, see Figure 1) as done in a pre-clinical study for epicardial PEF ablation [1]. We evaluated two pulse intervals: 100 µs and 10 µs (see Figure 3). To model all these configurations, an electrical boundary condition (PEF pulse trains) was applied at the metal active electrode, while ϕ = 0 V was set at the dispersive electrode. Electric current was set to be zero in all the outer surfaces of the model except the surface corresponding to the dispersive electrode.

For thermal boundary conditions, thermal convection coefficients were applied at the air-electrode interface (20 W/m^2^ K, 21 °C ambient temperature) [23] and at the myocardium- blood interface to simulate blood circulation (1417 W/m^2^K, 37 °C blood temperature) calculated under conditions of high blood flow (24.4 cm/s), as detailed in [24,25]. In the case of having the artery without stent, we simplified the situation by assuming a constant velocity of 0.5 m/s, which is half of the peak value reported in [26]. This corresponded with a convection coefficient of 63.19 W/m^2^ K calculated as in [24,25]. To perform these calculations of convection coefficients, we considered the viscosity of the blood to be 2.1 × 10^−3^ kg/(m·s) [25].

### 2.5. Analysed Outcomes

Simulations were conducted to assess if the presence of the stent affects the E-field distribution during epicardial PEF ablation. We modelled different scenarios: epicardial fat layer without an artery, and an artery with and without the stent. We used the 1000 V/cm isoline to assess and estimate ‘the PEF-zone’ size (maximum width and depth) as done in [27]. The electric field threshold value of 1000 V/cm was recently reported for PEF-induced irreversible damage in the myocardium [28]. Temperature distribution was computed to assess possible undesirable thermal side effects, particularly near the ablation electrode and stent. Transient simulations time was extended 10 ms after the last PEF pulse to check if thermal latency provoked a temperature increment during that period.

## 3. Results

### 3.1. Electric Field Distribution

**Figure 4:**
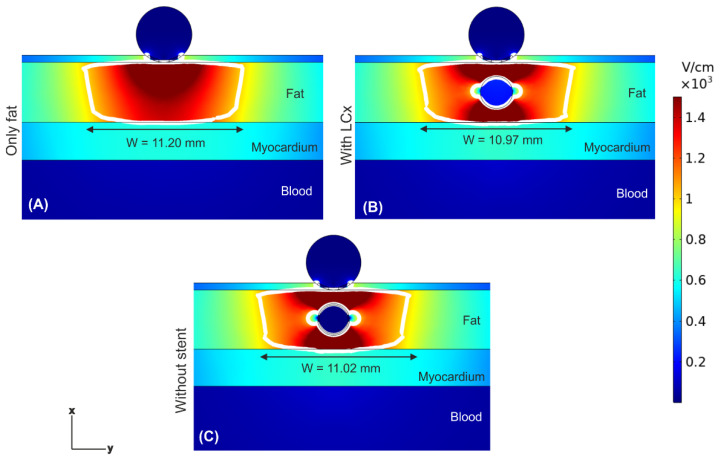
Electric field distributions after a train of ten PEF pulses (100 μs between them) for a distance of 1 mm between ablation electrode and artery/stent. Different scenarios were compared: (**A**) epicardial fat layer without artery, (**B**) artery without stent, and (**C**) artery with stent. The white contour corresponds to the 1000 V/cm electric field isoline and defines the PEF-zone.

**Figure 5:**
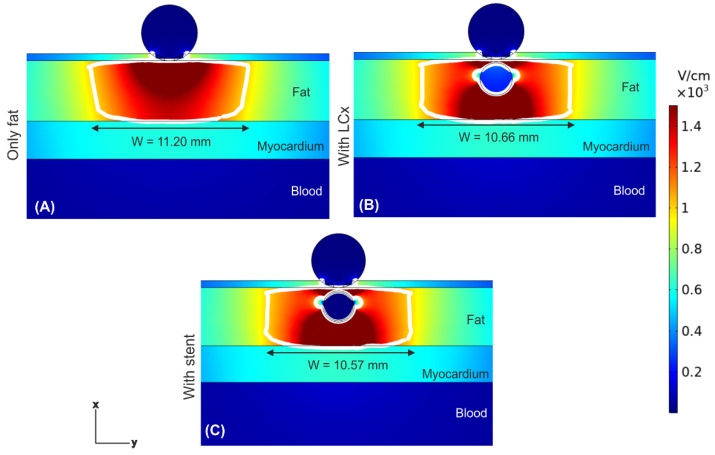
Electric field distributions after a train of ten PEF pulses (100 μs between them) for a distance of 0.25 mm between ablation electrode and artery/stent. Different scenarios were compared: (**A**) epicardial fat layer without artery, (**B**) artery without stent, and (**C**) artery with stent. The white contour corresponds to the 1000 V/cm electric field isoline and defines the PEF-zone.

Figure 4 shows the electric field distributions at the end of 10 PEF pulses (100 μs between pulses) for three scenarios: in the absence of a coronary artery, in the presence of a coronary artery, and in the presence of an artery with a metal stent inside (at 1 mm from the ablation electrode). The most important finding was that the presence of the artery under the ablation electrode (whether metal stent was present or absent) provoked a noticeable distortion of the E-field distribution, with hot spots (higher E-field values) in the front and rear of the artery, and cold spots (lower E-field values) on the sides of the artery. Despite of this distortion, the PEF-zone (white line) was confined within the fat for any case, not affecting the underlying myocardium. The PEF-zone width barely changed, from 10.97 mm when only the artery was present (Figure 4B) to 11.20 mm in the case without artery (Figure 4A). The values of E-field inside the artery without a stent were much lower than in the surrounding adipose tissue (only 218 V/cm), and negligible when the stent was present (only 2.7 × 10^−4^ V/cm). This same electrical performance was observed when the distance between ablation electrode and artery + stent was reduced to 0.25 mm (see Figure 5); simply the distribution of the aforementioned hot and cold spots was altered, being less symmetrical around the artery.

### 3.2. Temperature Distributions

**Figure 6:**
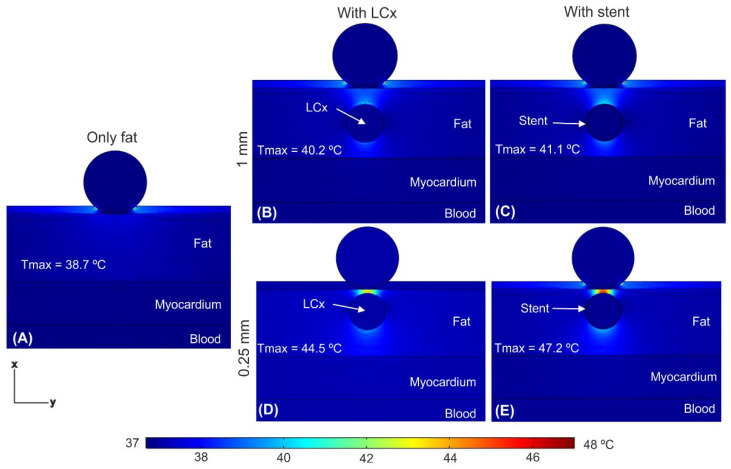
Temperature distributions just after applying a pulse train with an interval between pulses of 100 μs comparing different conditions: epicardial fat layer without artery (**A**), artery at 1 mm from the ablation device without (**B**) and with (**C**) metal stent, and artery at 0.25 mm without (**D**) and with (**E**) with metal stent.

**Figure 7:**
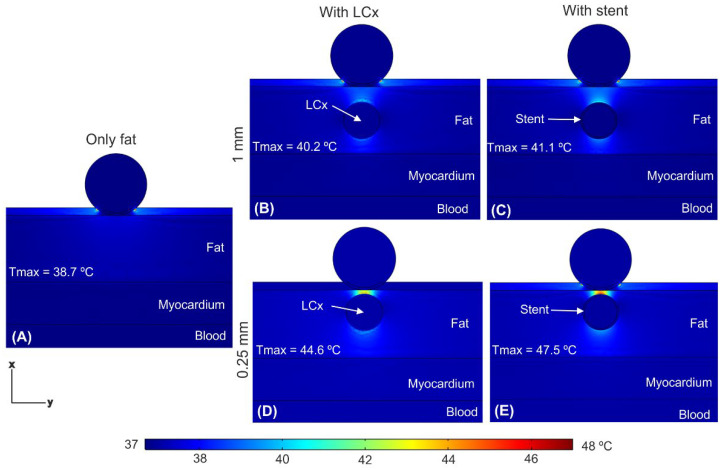
Temperature distributions just after applying a pulse train with time between pulses of 10 μs (see Figure 3B) comparing different conditions: (**A**) epicardial fat layer without LCx, the LCx at 1 mm from the ablation device without (**B**) and with (**C**) with the metal stent, and the LCx at 0.25 mm without (**D**) and with (**E**) the metal stent.

Figure 6 shows the temperature distributions just after applying a train of ten 100 μs pulses with an interval of 100 μs between them (as shown in Figure 3A) and under three scenarios: in the absence of a coronary artery, in the presence of a coronary artery, and in the presence of an artery with a stent. We confirmed that the tissue temperature peaked just at the end of the last PEF pulse, and not afterwards. The presence of the artery (with or without stent) caused a distortion in temperature distribution, specifically the appearance of two hot spots which was more prominent for 0.25 mm distance (Figure 6D,E): one quite prominent in the front (between the electrode and the artery), with a peak temperature of 44.5 °C without stent and 47.2 °C with stent, and a less important one in the rear (between the artery and the myocardium). The presence of the stent provoked a slight increment in the T_max_, which was higher when the stent was closer to the ablation device: 0.9 °C at 1 mm and 2.7 °C at 0.25 mm (see Figure 6B–E). Shortening the time between pulses to 10 μs hardly caused an increase in T_max_ (see Figure 7) compared to the case of 100 μs, regardless the stent-electrode distance (differences ≤ 0.3 °C).

## 4. Discussion

### 4.1. Main Findings

Although a recent study [11] suggested that epicardial pulsed electric field (PEF) ablation of GP produce minimal risk of electrical damage to the adjacent organs (lungs and oesophagus), the effect of metal intracoronary stents in the vicinity of the ablation electrode has not been assessed yet. Metal stents have been demonstrated to channel the electric current, resulting in an increase in the temperature during irreversible electroporation (IRE) in porcine liver and pancreas with a metal biliary stent [6,29]. To date, there is no experimental or computational study that has evaluated the presence of a metal intracoronary stent during cardiac PEF ablation. As far as we know, our study is the first computational model-based study for epicardial PEF ablation in which the presence of a stent placed near the ablation electrode was assessed in terms of E-field and thermal distributions. The main findings of the study are:

(1) The presence of the coronary artery near the ablation electrode (with or without a stent) distorts the E-field distribution, creating hot spots (higher E-field values) in the front and rear of the artery, and cold spots (lower E-field values).
(2) The value of E-field inside the coronary artery is very low (~200 V/cm), and almost zero in case with a metal stent.
(3) Despite this distortion, the PEF-zone contour (assessed as the isoline of 1000 V/cm) is almost identical with and without artery/stent, remaining almost completely confined within the fat layer in any case.
(4) The mentioned hot spots of E-field translate into a moderate temperature increase (<48 °C) in the area between artery and electrode.
(5) The thermal distribution is similar for pulses intervals of 10 and 100 μs.

In a previous computational modelling study, we found that due to the higher electrical conductivity of the neuronal tissue (GPs), compared to the surrounding adipose tissue, the E-field distribution distorted, resulting in a decrease in the local E-field value at the GP, which could compromise the efficacy of PEF ablation [4]. What we have learned from the current study has clinical implications in terms of safety, rather than efficacy. In particular, we observed that not only the presence of a nearby metal stent can alter the E-field distribution, but also the presence of the coronary artery alone can do this, due to the electrical conductivity of blood being much greater than that of fat (0.748 vs. 0.0438 S/m). This behaviour associated with tissues of very different electrical conductivities was already observed in a modelling study for electroporation of liver tumour nodules [30].

This E-field distortion was sufficiently pronounced that, in the case of very short distances (0.25 mm), it created points with a very high local E-field value which increased moderately the tissue temperature up to ~44 °C. Furthermore, this phenomenon was more pronounced when an intracoronary stent was present, reaching up to 47.5 °C. Since these values are below the lethal isotherm (~55 °C) [31], they should be considered as moderate hyperthermia without associated irreversible thermal damage (i.e., necrosis). Note that we simulated the worst-case scenario in which the interval between pulses is extremely short (10 and 100 μs), so in a clinical scenario where this interval may be longer in duration (i.e., 1 s) [1] no thermal side effect is expected.

Although the presence of a coronary artery (with or without a stent) might distort the E-field and even provoke side effects, it is interesting to note that the PEF-zone size hardly changed compared to the case without artery, which suggests that the efficacy (assessed in terms of the extension of the PEF-zone) would not be affected by the presence of the artery/stent. It is also interesting to point out that, despite the proximity of coronary artery to the ablation electrode, the E-field inside it remained at very low values (~200 V/cm), possibly below the threshold of damage by PEF in the blood, while in the case with the stent, the E-field was practically zero due to the Faraday cage effect.

Additionally, we found that the tissue temperature peaked just at the end of the PEF pulses, and not later. In other words, there does not seem to be a significant thermal latency effect, unlike what is observed in radiofrequency ablation using high-power very short-duration pulses [32], where the temperature peak can occur several seconds after power has been ceased.

### 4.2. Limitations

The model was 2D, which implies that we assumed an infinitely long electrode. This means that the results only reflect the behaviour of the electric field right in the middle of the electrode, and not at its ends, where an edge effect could be found. Despite this, the results are relevant and have clinical implications because the model assumes the worst scenario in which the ablation electrode is exactly positioned at the artery. In addition, our model assumed values of pre- and post-electroporation electrical conductivities based on values for different frequencies (low and high, respectively). Although this is common practice in computational models of electroporation, and moreover makes sense from a physical point of view, our results should be taken with caution until specific experimental measurements of these electrical conductivities are available under the same conditions that occur in PEF epicardial ablation and for each involved specific tissue.

## 5. Conclusions

In this study we built a numerical model to assess how the E-field distribution is affected by the presence of a metal intracoronary stent, along with the possible thermal side effects during PEF ablation. Our findings suggest that the presence of a metal intracoronary stent near the ablation device during epicardial PEF ablation simply ‘amplifies’ the E-field distortion already caused by the presence of a nearby coronary artery. This distortion may involve moderate heating (<48 °C) between the artery and the ablation electrode without associated thermal damage. Our results encourage the execution of future experimental studies that confirm these findings.

## Figures and Tables

**Table 1:** Electrical and thermal properties of the model elements [16,17,18,19,20].

Element/Material	*σ*_0_ (S/m)	*σ*_1_ (S/m)	*k* (W/m·K)	*ρ* (kg/m^3^)	*c* (J/kg·K)
Electrode/Pt-Ir	4.6 × 10^6^		71	21,500	132
Catheter/Polyurethane	10^−5^		23	1440	1050
Stent/Stainless Steel	7.4 × 10^6^		15	8000	480
Saline	1.392		0.628	980	4184
Fat	0.0377	0.0438	0.21	911	2348
Heart/myocardium	0.0537	0.281	0.56	1081	3686
Blood	0.7	0.748	0.52	1050	3617

*σ*: Electrical conductivity (*σ*_0_ and *σ*_1_ being the pre- and post-electroporation electrical conductivity values, respectively); *k*: thermal conductivity; *ρ:* density; *c*: specific heat.

## Data Availability

Not applicable.

## References

[1] Padmanabhan D., Naksuk N., Killu A.K., Kapa S., Witt C., Sugrue A., DeSimone C.V., Madhavan M., de Groot J.R., O’Brien B. (2019). Electroporation of epicardial autonomic ganglia: Safety and efficacy in medium-term canine models. J. Cardiovasc. Electrophysiol..

[2] Madhavan M., Venkatachalam K.L., Swale M.J., Desimone C.V., Gard J.J., Johnson S.B., Suddendorf S.H., Mikell S.B., Ladewig D.J., Nosbush T.G. (2016). Novel Percutaneous Epicardial Autonomic Modulation in the Canine for Atrial Fibrillation: Results of an Efficacy and Safety Study. Pacing Clin. Electrophysiol..

[3] Avazzadeh S., McBride S., O’Brien B., Coffey K., Elahi A., O’Halloran M., Soo A., Quinlan L.R. (2020). Ganglionated Plexi Ablation for the Treatment of Atrial Fibrillation. J. Clin. Med..

[4] González-Suárez A., O’Brien B., O’Halloran M., Elahi A. (2022). Pulsed electric field ablation of epicardial autonomic ganglia: Computer analysis of monopolar electric field across the tissues involved. Bioengineering.

[5] Hogenes A.M., Slump C.H., Te Riet O.G., Scholten G.A., Meijerink M.R., Fütterer J.J., van Laarhoven C.J.H.M., Overduin C.G., Stommel M.W.J. (2020). Effect of irreversible electroporation parameters and the presence of a metal stent on the electric field line pattern. Sci. Rep..

[6] Scheffer H.J., Vogel J.A., van den Bos W., Neal R.E., van Lienden K.P., Besselink M.G., van Gemert M.J., van der Geld C.W., Meijerink M.R., Klaessens J.H. (2016). The Influence of a Metal Stent on the Distribution of Thermal Energy during Irreversible Electroporation. PLoS ONE.

[7] Scheffer H.J., Vogel J.A., van den Bos W., Meijerink M.R., Besselink M.G., Verdaasdonk R.M., Klaessens J., van der Geld C.W., van Gemert M.J. (2015). Comment to: Månsson C, Nilsson A, Karlson B-M. Severe complications with irreversible electroporation of the pancreas in the presence of a metallic stent: A warning of a procedure that never should be performed. Acta Radiol. Open.

[8] Iasiello M., Andreozzi A., Bianco N., Vafai K. (2020). The porous media theory applied to radiofrequency catheter ablation. Int. J. Numer. Methods Heat Fluid Flow.

[9] Wang S., Vafai K. (2013). Analysis of the effect of stent emplacement on LDL transport within an artery. Int. J. Heat Mass. Transf..

[10] Sánchez-Quintana D., López-Mínguez J.R., Macías Y., Cabrera J.A., Saremi F. (2014). Left atrial anatomy relevant to catheter ablation. Cardiol. Res. Pract..

[11] González-Suárez A., Irastorza R.M., Deane S., O’Brien B., O’Halloran M., Elahi A. (2022). Full torso and limited-domain computer model for epicardial pulsed electric field ablation. Comput. Methods Programs Biomed..

[12] Yokokawa M., Sundaram B., Garg A., Stojanovska J., Oral H., Morady F., Chugh A. (2011). Impact of mitral isthmus anatomy on the likelihood of achieving linear block in patients undergoing catheter ablation of persistent atrial fibrillation. Heart Rhythm..

[13] Saito A., Dai Z., Ono M., Kanie T., Takaoka Y., Mizuno A., Komiyama N., Asano T. (2022). The relationship between coronary stent strut thickness and the incidences of clinical outcomes after drug-eluting stent implantation: A systematic review and meta-regression analysis. Catheter. Cardiovasc. Interv..

[14] Sheehan M.C., Srimathveeravalli G., Prakash P., Srimathveeravalli G. (2021). Pulsed electric fields. Principles and Technologies for Electromagnetic Based Therapies. Principles and Technologies for Electromagnetic Energy Based Therapies.

[15] Berjano E.J. (2006). Theoretical modeling for radiofrequency ablation: State-of-the-art and challenges for the future. Biomed. Eng. Online.

[16] Pérez J.J., Ewertowska E., Berjano E. (2020). Computer Modeling for Radiofrequency Bipolar Ablation Inside Ducts and Vessels: Relation Between Pullback Speed and Impedance Progress. Lasers Surg. Med..

[17] Hasgall P.A., Di Gennaro F., Baumgartner C., Neufeld E., Lloyd B., Gosselin M.C., Payne D., Klingenböck A., Kuster N. It Is Database for Thermal and Electromagnetic Parameters of Biological Tissues, Version 4.1, 22 February 2022. https://itis.swiss/virtual-population/tissue-properties/overview/.

[18] Gabriel C., Peyman A., Grant E.H. (2009). Electrical conductivity of tissue at frequencies below 1 MHz. Phys. Med. Biol..

[19] Gopalakrishnan J.A. (2002). A mathematical model for irrigated epicardial radiofrequency ablation. Ann. Biomed. Eng..

[20] Berjano E.J., Burdío F., Navarro A.C., Burdío J.M., Güemes A., Aldana O., Ros P., Sousa R., Lozano R., Tejero E. (2006). Improved perfusion system for bipolar radiofrequency ablation of liver: Preliminary findings from a computer modelling study. Physiol. Meas..

[21] Sel D., Cukjati D., Batiuskaite D., Slivnik T., Mir L.M., Miklavcic D. (2005). Sequential finite element model of tissue electropermeabilization. IEEE Trans. Biomed. Eng..

[22] Castellví Q., Mecadal B., Moll X., Fondevila D., Andaluz A., Ivorra A. (2018). Avoiding neuromuscular stimulation in liver irreversible electroporation using radiofrequency elect ric fields. Phys. Med. Biol..

[23] Suárez A.G., Hornero F., Berjano E. (2010). Mathematical modeling of epicardial RF ablation of atrial tissue with overlying epicardial fat. Open Biomed. Eng. J..

[24] Tungjitkusolmun S., Vorperian V.R., Bhavaraju N., Cao H., Tsai J.Z., Webster J.G. (2001). Guidelines for predicting lesion size at common endocardial locations during radio-frequency ablation. IEEE Trans. Biomed. Eng..

[25] González-Suárez A., Berjano E. (2016). Comparative analysys of different methods of modeling the thermal effect of circulating blood flow during RF cardiac ablation. IEEE Trans. Biomed. Eng..

[26] Anjaneyulu A., Raghu K., Chandramukhi S., Satyajit G.M., Arramraja S., Raghavaraju P., Krishnamraju P., Somaraju B. (2008). Evaluation of left main coronary artery stenosis by transthoracic echocardiography. J. Am. Soc. Echocardiogr..

[27] Stewart M.T., Haines D.E., Miklavčič D., Kos B., Kirchhof N., Barka N., Mattison L., Martien M., Onal B., Howard B. (2021). Safety and chronic lesion characterization of pulsed field ablation in a Porcine model. J. Cardiovasc. Electrophysiol..

[28] Avazzadeh S., O’Brien B., Coffey K., O’Halloran M., Keane D., Quinlan L.R. (2021). Establishing irreversible electroporation electric field potential threshold in a suspension in vitro model for cardiac and neuronal cells. J. Clin. Med..

[29] Agnass P., van Veldhuisen E., Vogel J.A., Kok H.P., de Keijzer M.J., Schooneveldt G., de Haan L.R., Klaessens J.H., Scheffer H.J., Meijerink M.R. (2020). Thermodynamic profiling during irreversible electroporation in porcine liver and pancreas: A case study series. J. Clin. Transl. Res..

[30] Castellví Q., Sánchez-Velázquez P., Berjano E., Burdío F., Ivorra A., Lacković I., Vasic D. (2015). Selective Electroporation of Liver Tumor Nodules by Means of Hypersaline Infusion: A Feasibility Study. IFMBE Proceedings, Proceedings of the 6th European Conference of the International Federation for Medical and Biological Engineering, Dubrovnik, Croatia, 7–11 September 2014.

[31] Haines D.E. (2011). Letter by Haines regarding article: Direct measurement of the lethal isotherm for radiofrequency ablation of myocardial tissue. Circ. Arrhythm. Electrophysiol..

[32] Irastorza R.M., d’Avila A., Berjano E. (2018). Thermal latency adds to lesion depth after application of high-power short-duration radiofrequency energy: Results of a computer-modeling study. J. Cardiovasc. Electrophysiol..

